# Outcomes following general anaesthesia in children with hypertrophic cardiomyopathy

**DOI:** 10.1136/archdischild-2018-315366

**Published:** 2018-11-09

**Authors:** Gabrielle Norrish, Natalie Forshaw, Colleen Woo, Mary Claire Avanis, Ella Field, Elena Cervi, Akane Iguchi, Juan Pablo Kaski

**Affiliations:** 1 Centre for Inherited Cardiovascular Diseases, Great Ormond Street Hospital, London, UK; 2 Institute of Cardiovascular Sciences, University College London, London, UK; 3 Department of Anaesthesia, Great Ormond Street Hospital, London, UK

**Keywords:** cardiology, anaesthetics

## Abstract

**Background:**

Children with hypertrophic cardiomyopathy (HCM) have historically been considered to be high-risk candidates for general anaesthesia (GA), but there is currently a paucity of evidence regarding the safety of anaesthesia and perioperative outcomes in this population.

**Methods:**

Clinical features and outcomes of all paediatric patients (<18 years) with HCM undergoing GA between 2000 and 2016 were reviewed.

**Results:**

86 patients (median 12.4 years (IQR 6.5, 14.9)) underwent 164 separate GA procedures. Aetiology included non-syndromic disease (n=44, 56%), malformation syndromes (n=22, 26%), inborn error of metabolism (n=10, 12%) and neuromuscular disease (n=4, 5%). At the time of GA, mean maximal wall thickness (MWT) on echocardiography was 19 mm (SD±8 mm), 23 (14%) patients had severe left ventricular hypertrophy (MWT>30 mm) and 35 (21%) patients had a haemodynamically significant left ventricular outflow tract (LVOT) gradient (>50 mm Hg). The majority (n=143, 87%) had no perioperative complications. 20 (12%) patients had minor perioperative complications: bradycardia (n=4), hypotension (n=15) or transient ST segment changes (n=1). One (0.6% of GA procedures) patient experienced a cardiac arrest during anaesthetic induction with death occurring 3 days later. Clinical parameters (including age, MWT, LVOT gradient, systolic and diastolic dysfunction) were not associated with an increased risk of complications

**Conclusions:**

This is the largest published series to date of paediatric patients with HCM undergoing GA, which demonstrates that, in an expert centre, patients can be anaesthetised with a relatively low perianaesthetic mortality (0.6%) and prevalence of minor complications (12%). Future studies are required to systematically identify clinical features that may predict anaesthetic risk.

**What is already known on this topic?**

- Children with hypertrophic cardiomyopathy (HCM) have historically been considered to be high-risk candidates for general anaesthesia.
- There is currently a paucity of evidence regarding the safety of anaesthesia and perioperative outcomes in this population.

**What this study adds?**

- In an expert centre, paediatric patients with HCM can be anaesthetised with a low perianaesthetic mortality (0.6%) and low prevalence of minor complications (12%).
- Future studies are required to systematically identify clinical features that may predict increased anaesthetic risk.

## Background

Hypertrophic cardiomyopathy (HCM) is the second most common cause of cardiomyopathy during childhood, with an estimated annual incidence of 0.24–0.47 per 100 000.^1–3^ The majority of disease is caused by mutations in the cardiac sarcomere protein genes,^4^ however aetiology is heterogeneous and also includes malformation syndromes, inborn errors of metabolism and neuromuscular diseases. Phenotypic expression is highly variable, but can include severe left ventricular hypertrophy (LVH), dynamic left ventricular outflow tract obstruction (LVOTO), impaired diastolic function and a predisposition to ventricular arrhythmias. The dynamic nature of LVOTO in HCM, caused by systolic anterior motion of the mitral valve, means that gradients can be exacerbated by changes in heart rate, heart rhythm, preload and/or afterload, with resulting haemodynamic instability. Although global systolic function is typically preserved, patients with HCM commonly have reduced LV compliance and diastolic dysfunction and are reliant on preload to maintain cardiac output. Finally, the hypertrophied left ventricle has an increased myocardial oxygen demand, resulting in a higher risk of coronary blood flow insufficiency and myocardial ischaemia. Given these characteristics, children with HCM have historically been viewed as high-risk general anaesthesia (GA) candidates. Perioperative outcomes of adult patients with HCM have been reported in the literature, although often with conflicting findings. However, there is currently a lack of evidence regarding the optimal perioperative management and safety of GA in the paediatric HCM population. The aim of this study was to determine the safety and outcomes of paediatric patients with HCM undergoing GA in a specialised centre.

## Patients and methods

### Patients

Clinical cardiac and anaesthetic data from children with HCM undergoing GA under the age of 18 years at Great Ormond Street Hospital between 2000 and 2016 were reviewed retrospectively. Patients were identified from anaesthetic records and a clinical database. All patients fulfilled conventional diagnostic criteria for HCM (maximal left ventricle wall thickness greater than 2 SDs above the predicted body surface area (BSA)-corrected mean in the absence of abnormal loading conditions).^5 6^ Patients were not included in this series if they underwent anaesthesia for cardiac surgery requiring cardiopulmonary bypass; had a ventricular assist device in situ; or were after heart transplantation. When patients had more than one anaesthetic, data were collected from all episodes, with descriptive demographic data obtained from the first anaesthetic, and clinical and perioperative data obtained relevant to each episode of anaesthesia.

### Data collection

Anonymised, non-invasive clinical information from the time of anaesthetic was collected, including: demographics; aetiology; pedigree; symptoms; and two-dimensional (2D) transthoracic echocardiography data. Aetiology was classified as non-syndromic in the absence of a diagnosis of a RASopathy syndrome (Noonan or other malformation syndrome), neuromuscular disease (including Friedreich’s ataxia) or inborn error of metabolism.

Echocardiographic measurements were made according to current guidelines.^7 8^ Specifically, maximum LV wall thickness (MLVWT) was defined as the greatest thickness in any single segment at end diastole as measured by 2D echocardiography. Perioperative information collected included: details of procedure performed; anaesthetic management; intraoperative pharmacological and physiological data; postoperative care; and complications occurring intraoperatively or within 24 hours of anaesthesia. Hypotension, tachycardia and bradycardia were defined as values more than 30% above or below patient’s own baseline or those outside age-specific normal values.

Major complications were defined a priori as perioperative mortality, myocardial infarction (MI), stroke or congestive cardiac failure (CCF). Minor complications were defined a priori as a transient disturbance in blood pressure, heart rate or arrhythmias requiring treatment, or the need for an unplanned intensive care admission.

### Definitions

Severe LVH was defined as MLVWT≥30 mm in any myocardial segment.^6^ Impaired LV systolic function was defined as LV fractional shortening <25%, as measured in parasternal long or short axis (2D or M mode) or ejection fraction <50% using Simpson’s biplane volumetric method.^7^ LV diastolic dysfunction was assessed to be present if two out of the four variables used to assess diastolic function were out of normal range for age and BSA (annular E′ velocity, average E/E′ ratio, LA volume and peak TR velocity).^8^ LVOTO was defined as an instantaneous peak Doppler LVOT pressure gradient ≥30 mm Hg at rest. A haemodynamically significant gradient was considered to be an instantaneous peak Doppler gradient ≥50 mm Hg.^9^ Non-sustained ventricular tachycardia (NSVT) was defined as three or more consecutive ventricular beats at a rate of greater than 120 beats/min with a duration of less than 30 s on ambulatory ECG recordings.^5^

### Statistical analysis

All statistical analyses were performed using STATA (Stata Statistical Software: Release 14. College Station, TX: StataCorp). BSA was calculated from height and weight.^10^ Normally distributed continuous variables are described as mean±SD with two group comparisons conducted using Student’s t-test. Skewed data are described as median (IQR) with two group comparisons performed using Wilcoxon rank-sum. To determine the association between relevant predictors and a composite outcome of primary or secondary outcomes, univariate analysis was performed using Χ^2^ test or Fisher’s exact test. To account for multiple comparisons, the Bonferroni correction was applied, accepting a p value <0.05 as significant for all analyses.

## Results

### Demographics

Eighty-six eligible patients with HCM under the age of 18 underwent 164 separate general anaesthetics between 2000 and 2016. Seventy-eight (90%) patients underwent more than one anaesthetic. Forty-eight (56%) patients had non-syndromic HCM, of whom 22 (46%) had undergone genetic testing. Pathogenic mutations in sarcomeric protein genes were identified in 15 patients (68% of those tested). Twenty-two (26%) patients had a RASopathy syndrome, 10 (12%) an inborn error of metabolism and 4 (5%) a neuromuscular disorder. Data on baseline clinical characteristics and comorbidities are shown in table 1. Four patients had a history of an out-of-hospital arrest. Thirteen (15%) patients had a history of NSVT on ambulatory ECG monitoring.

**Table 1:** Baseline clinical characteristics

	n (%)
Male gender	50 (58)
Age, years (median (range))	12.4 (0–18.4)
Weight, kg (median (range))	38.6 (2–101)
Cardiac medications	
Beta-blockers	91 (55)
Disopyramide	38 (23)
Calcium channel blockers	16 (10)
Amiodarone	10 (6)
Comorbidities (n)	
ENT	
OSA	3
Orthopaedic	
Scoliosis	4
Polyarthralgia	1
Respiratory	
Asthma	4
Congenital cardiac disease	
VSD	1
Aortic stenosis	1
Neurology	
Epilepsy	1
Autistic spectrum disorder	3
Developmental delay (acquired/congenital)	3
Hydrocephalus	1
Metabolic	
Diabetes mellitus	2
Renal	
Polycystic kidney	1

Data expressed as number (%). Total number of patients is 86 unless otherwise stated.

ENT, ears, nose and throat; OSA, obstructive sleep apnoea; VSD, ventricular septal defect.

### Clinical phenotype at time of anaesthesia

Mean MLVWT at the time of anaesthesia was 19 mm (SD 8.3 mm, range 4–49 mm). The distribution of hypertrophy was asymmetric in 106 (68%) patients, concentric in 47 (30%) and biventricular in 3 (2%). Twenty-three (14%) patients had severe LVH. Of 47 (31%) patients with LVOTO at rest, 35 (21%) had a haemodynamically significant gradient (>50 mm Hg) and one had severe outflow tract obstruction (>90 mm Hg). Eighty-seven (53%) patients had echocardiographic evidence of impaired LV diastolic performance and 10 (6%) patients had impaired LV systolic function. Twenty-four (15%) patients had an implantable cardioverter defibrillator (ICD) in situ at the time of anaesthesia. One hundred and five (64%) patients were on medical therapy for symptom control. Forty-six (28%) patients were on more than one medication (table 1).

### Indication for general anaesthetic

One hundred and fifty (91%) general anaesthetics were for elective procedures. The indication for anaesthetic is shown in table 2.

**Table 2:** Indication for general anaesthetic

	n (%)
Cardiac	87 (53)
Implantation/threshold testing of ICD	56
Diagnostic cardiac catheterisation	7
Interventional cardiac catheterisation	1
EPS	9
Pacemaker implantation	8
Loop recorder insertion	4
Transoesophageal echocardiogram	2
General surgical	11 (7)
Gastrointestinal	7
Biopsy/excision	4
Radiology	26 (16)
Diagnostic	9
Interventional	17
Orthopaedic	12 (8)
Ears, nose and throat	8 (5)
Dental	8 (5)
Plastics	2 (1)
Neurosurgery	2 (1)
Urology	7 (4)
Respiratory	1 (<1)

Data expressed as number (%). Total number of general anaesthesia (GA) procedures is 164.

EPS, electrophysiology study; ICD, implantable cardioverter defibrillator.

### Perioperative anaesthetic management

Details on perioperative anaesthetic management are summarised in table 3. The lead anaesthetic provider was a consultant cardiac anaesthetist in 75 (46%), consultant non-cardiac anaesthetist in 76 (46%) and a senior anaesthetic trainee doctor in 13 (8%). One hundred (61%) patients had an intravenous induction; phenylephrine was used empirically at induction in 8 (5%) patients with no subsequent recorded hypotension. In 153 (93%) patients, anaesthesia was maintained with volatile agents. All patients had intraoperative non-invasive cardiorespiratory monitoring, 47 (29%) patients had invasive arterial blood pressure monitoring and 14 (9%) had central venous pressure monitoring.

**Table 3:** Perianaesthetic management

Anaesthetic detail	n (%)
Lead anaesthetic provider	
Consultant—cardiac	75 (46)
Consultant—non-cardiac	76 (46)
Senior trainee doctor	13 (8)
Anxiolytic premedication used	34 (21)
Mode of induction	
Gaseous	60 (37)
Sevoflurane	60
Intravenous	100 (61)
Propofol and opioid	40
Etomidate and opioid	33
Ketamine and opioid	5
Propofol only	15
Other	7
Mixed (intravenous+gas)	4 (2)
Airway management	
Endotracheal intubation	142 (87)
Laryngeal mask airway	19 (12)
Other	3 (2)
Maintenance	
Volatile	153 (93)
Total intravenous anaesthesia	9 (5)
Not specified	2 (1)
Vasopressor agent (n=17, 10%)	
Phenylephrine	14 (9)
Norepinephrine	3 (1)

Data expressed as number (%).

### Perioperative complications

Of a total of 164 anaesthetics in children with HCM, 143 (87%) had no perioperative complications. Twenty (12%) patients had minor perioperative complications: 15 patients had intraoperative hypotension requiring treatment; 4 had intraoperative bradycardia requiring treatment; and 1 had transient ST segment changes not requiring treatment. One (0.6%) patient had a major complication, with cardiac arrest occurring after induction of GA requiring extracorporeal-cardiopulmonary resuscitation and death occurring 3 days later. This patient had familial HCM with a severe phenotype: asymmetric hypertrophy (maximal wall thickness (MWT) 20 mm); LVOTO at rest (55 mm Hg) managed with medical therapy (atenolol and disopyramide) and short atrio-ventricular (AV) delay dual-chamber pacing; and evidence of LV diastolic impairment with left atrial dilatation. A primary prevention ICD had previously been implanted following syncopal events and the detection of NSVT on ambulatory ECG monitoring. No clinical parameters were associated with an increased risk of reaching the composite outcome of any postoperative complication (table 4).

**Table 4:** Comparison of patients by the presence of complications

	Complication	No complication	P values
Age, years (median (IQR))	10.74 (0.23–16.6)	10.4 (0.01–18.43)	>0.999
Weight, kg (median (range))	39.6 (5.6–77)	39.6 (2–101.4)	>0.999
Emergency procedure, n%	10% (n=2/21)	8% (n=11/140)	>0.999*
ASH distribution, n%	65% (n=13/20)	67% (n=91/136)	>0.999
Systolic dysfunction, n%	20% (n=4/20)	4% (n=6/136)	0.064
Diastolic dysfunction, n%	89% (n=16/18)	74% (n=71/96)	>0.999
LVOT gradient, n%>50 mm Hg	24% (n=5/21)	17% (n=23/136)	>0.999*
MLVWT>30 mm, n%	15% (n=3/20)	16% (n=20/129)	>0.999*

Data expressed as % (n=proportion of patients with available data). Comparisons performed using χ^2^ test unless otherwise stated.

*Fisher’s exact test.

ASH, asymmetric septal hypertrophy; LVOT, left ventricular outflow tract; MLVWT, maximal left ventricular wall thickness.

### Postoperative course

Postoperative recovery took place on a cardiology ward for 91 (55%) patients, including 10 (6%) who had undergone non-cardiac procedures. Seventeen (10%) patients were managed postoperatively on intensive care, including two patients who had an unplanned admission: one following a failed extubation, and one after cardiac arrest requiring extracorporeal membrane oxygenator support (as described above). Length of stay ranged from 0 to 21 days (median 1 day); 104 (63%) procedures were completed as day case admissions and did not require an overnight stay.

## Discussion

To our knowledge, this is the largest study of perianaesthetic outcomes in children with HCM to date. The results demonstrate that, in a heterogeneous cohort of patients with HCM having a GA at an experienced centre, the majority of patients (n=143, 87%) had no perioperative complications and two-thirds of procedures were performed as day cases requiring no overnight stay. Minor perioperative complications were seen in 12% of patients but there was 1 (0.6%) perioperative mortality in an adolescent with severe HCM.

### Comparison with previous literature

To date, no large cohort studies describing the safety of GA in paediatric HCM have been published and most of the literature consists of single-case reports. The largest published paediatric series reported the outcome of 129 patients with different cardiomyopathies undergoing anaesthesia in a single centre, of whom 50 had HCM.^11^ No deaths were reported among the patients with HCM, but the study was limited by a lack of detailed clinical description of patients. The scarcity of paediatric data means that expected outcomes for these patients are often extrapolated from adult studies, the findings of which have been conflicting. Early reports described a high frequency of perianaesthetic adverse events of up to 40% in adult patients with HCM.^12^ However, more recent studies describing larger populations of patients have reported a lower risk of cardiovascular adverse events, although the risk is nonetheless increased compared with controls.^13^ In the study by Dhillon and colleagues, 20% of patients with HCM reached a composite endpoint of death, MI, stroke or CCF; this was mostly driven by decompensated CCF and the absolute number of deaths, MI, or stroke was low and not significantly different from what was seen in an age-matched control group.^14^ The reported proportion of adult patients with HCM dying in the perioperative period in the literature ranges from 4% to 6.7%,^13 14^ which is higher than that seen in our cohort. This difference in mortality is unlikely to be explained by a milder disease phenotype. While the adult studies had a higher proportion of patients with LVOTO (53% vs 30.5%) and diastolic dysfunction (93% vs 55%), the absolute MWT and proportion of patients with a previous ventricular arrhythmia were not significantly different. The difference in mortality could be explained by the higher proportion of patients in the adult cohorts with additional comorbidities such as hypertension, diabetes and atrial fibrillation which are known to independently increase the risk of a GA.

### Identifying patients at high risk of adverse events

Although the prevalence of adverse events is lower than historically reported, the ability to identify patients at higher risk would help guide clinical management. However, no data currently exist to guide perianaesthetic risk stratification in paediatric HCM. In an adult cohort, the presence of LVOT obstruction at rest (gradient >30 mm Hg), a high American Society of Anesthesiologists risk score^15^ and intraoperative hypotension were associated with a higher risk of having an adverse event.^14^ Our ability to systematically investigate individual risk factors for an adverse perioperative outcome in this study was limited by low numbers of adverse events in this cohort. No single parameter, including MLVWT, LVOTO and diastolic impairment, was found to be associated with a perianaesthetic complication. However, it is likely that the combination of certain phenotypic features may confer a higher risk. Certainly, the single patient who experienced a major complication had severe disease with significant LVH, LVOTO, diastolic impairment and a history of ventricular arrhythmias. While larger studies are required to identify clinical features that may confer a higher anaesthetic risk, in clinical practice, it is essential that each individual patient is systematically assessed prior to a GA.

### Importance of expert centres

Childhood HCM is a rare disease and compared with adult HCM populations, the disease in childhood is more heterogeneous in terms of aetiology, symptoms and outcomes.^3 16 17^ The cohort of childhood patients with HCM reported here is representative of other cohorts described in population-based or registry studies.^1 2 18^ It includes patients with phenotypically severe disease as defined by severe LVH, LVOTO, diastolic impairment and previous ventricular arrhythmias. The results are therefore likely to be generalisable to the wider paediatric HCM population. However, this cohort is derived from a single centre with medical, surgical and anaesthetic expertise in managing patients with this heterogeneous disease. To allow for individualised perioperative planning, a multidisciplinary team meeting (including the paediatric HCM specialists and anaesthetic consultants) is held in advance of any procedure requiring a general anaesthetic. The low prevalence of adverse outcomes may not be replicable in non-specialist centres. It is important to note that early reports from adult cohorts, which reported a higher prevalence of adverse events, were indeed derived from non-specialist centres. This includes the largest cohort of patients with HCM undergoing GA to date (n=227), which identified patients from the US National Hospital Discharge Survey,^13^ a fifth of whom underwent GA procedures in community-based hospitals which may lack the expertise to manage these patients. This study reported an increased risk of death (OR 1.61) or MI in adults with HCM compared with age-matched controls, a finding that has not been replicated in subsequent cohort studies from specialist centres. The low prevalence of adverse outcomes reported here highlights the importance of developing expertise at a specialist centre.

### Limitations

This study is limited by problems inherent to retrospective studies, including missing or incomplete data. Additionally, although this is the largest cohort of paediatric patients with HCM undergoing a GA reported in the literature, the interpretation of the prevalence of complications is limited by the absence of age-matched controls. Previous studies have reported 30-day mortality of 1.1/10 000 (95% CI 0.4 to 2.6) in a non-selective cohort of children undergoing GA at a tertiary paediatric hospital.^19^ In contrast, in the largest study to date, assessing outcomes following non-cardiac surgery in children with congenital heart disease (CHD), higher postoperative mortality 3.9%–8.2% rates were described in patients with moderate (defined as repaired CHD with residual haemodynamic abnormality) and severe (including patients with ventricular dysfunction requiring medication or uncorrected cyanotic heart disease) CHD compared with controls (1.7%–1.2%).^20^ This suggests that, although children with HCM have a higher incidence of major complications following anaesthesia compared with the general population, the risk may be lower than in children with other cardiac disease. This series did not include patients undergoing cardiac surgery requiring cardiopulmonary bypass (CPB) as the interpretation of the a priori defined complications (in particular disturbances in blood pressure, heart rate or arrhythmias) would not be possible once CPB was established. Future studies investigating the outcome of children with HCM undergoing GA for procedures requiring CPB would be useful.

## Conclusion

This study reports the largest published series of patients with paediatric HCM undergoing GA and demonstrates a relatively low perianaesthetic mortality (0.6%) and prevalence of minor complications (12%). The findings suggest that, in an expert centre, children with HCM can be anaesthetised with a low risk of adverse events. Future studies are required to systematically identify clinical features that may predict anaesthetic risk.
